# Improvement of Ultrasound Image Quality Using Non-Local Means Noise-Reduction Approach for Precise Quality Control and Accurate Diagnosis of Thyroid Nodules

**DOI:** 10.3390/ijerph192113743

**Published:** 2022-10-22

**Authors:** Kyuseok Kim, Nuri Chon, Hyun-Woo Jeong, Youngjin Lee

**Affiliations:** 1 Department of Integrative Medicine, Major in Digital Healthcare, Yonsei University College of Medicine, Unju-ro, Gangman-gu, Seoul 06229, Korea; 2 Woori Yonsei Internal Medicine, 370, Anyang-ro, Manan-gu, Anyang-si 13991, Korea; 3 Department of Biomedical Engineering, Eulji University, 553, Sanseong-daero, Sujeong-gu, Seongnam-si 13135, Korea; 4 Department of Radiological Science, College of Health Science, Gachon University, 191, Hambakmoe-ro, Yeonsu-gu, Incheon 21936, Korea

**Keywords:** ultrasound quality control, accurate diagnosis of thyroid nodules, non-local means approach, noise reduction, quantitative evaluation of image quality

## Abstract

This study aimed to improve the quality of ultrasound images by modeling an algorithm using a non-local means (NLM) noise-reduction approach to achieve precise quality control and accurate diagnosis of thyroid nodules. An ATS-539 multipurpose phantom was used to scan the dynamic range and gray-scale measurement regions, which are most closely related to the noise level. A convex-type 3.5-MHz frequency probe is used for scanning according to ATS regulations. In addition, ultrasound images of human thyroid nodules were obtained using a linear probe. An algorithm based on the NLM noise-reduction approach was modeled based on the intensity and relative distance of adjacent pixels in the image, and conventional filtering methods for image quality improvement were designed as a comparison group. When the NLM algorithm was applied to the image, the contrast-to-noise ratio and coefficient of variation values improved by 28.62% and 19.54 times, respectively, compared with those of the noisy images. In addition, the image improvement efficiency of the NLM algorithm was superior to that of conventional filtering methods. Finally, the applicability of the NLM algorithm to human thyroid images using a high-frequency linear probe was validated. We demonstrated the efficiency of the proposed algorithm in ultrasound images and the possibility of capturing improved images in the dynamic range and gray-scale region for quality control parameters.

## 1. Introduction

This examination method can capture real-time images non-invasively and non-destructively; therefore, ultrasound imaging is actively used for the physical examination of the abdomen, thyroid gland, and breast [1,2,3]. An ultrasound examination is conducted to establish the cause of pain or inflammation. It may also aid in the early identification of tumors in patients without symptoms. It is often used to evaluate prenatal fetuses [4]. In addition, when performing a biopsy on multiple organs, ultrasonography can be safely and efficiently performed to monitor the ultrasound image in real time [5].

Recently, the use of ultrasound for the early diagnosis of cancer and other illnesses has rapidly increased. In Korea, the number of patients who underwent abdominal ultrasounds in 2019 was approximately 3.5 million; this number has increased exponentially since 2017 [6]. Compared with early lesion detection in relatively young patients, ultrasound examination for accurate lesion observation in elderly patients is crucial. Mahale et al. reported that ultrasound examination during health checkups significantly helps in the early detection of fatty liver and the accurate diagnosis of metabolic syndrome in asymptomatic patients [7]. With the increase in interest and number of ultrasound examinations, the importance of quality control of the device is also increasing. Management policies focusing on pre-testing are being implemented worldwide to reduce the number of unnecessary or inappropriate ultrasound examinations. The importance of quality control for accurate ultrasound device management is gradually increasing owing to this situation. Thus, the Korean Society of Radiology and the Korean Society of Ultrasound in Medicine developed a method that utilizes the ATS 539 multipurpose phantom as a standard for ultrasound image evaluation [8]. Quality control of the ultrasound imaging equipment is achieved by evaluating eight parameters according to medical device-related laws and enforcement regulations.

Although most ultrasound diagnostic devices pass the quality control criteria for the eight parameters, the image quality attributed to noise is declining. When an inappropriate ultrasound diagnostic device is used, the diagnosis accuracy decreases and the re-examination rate increases, which may cause a significant problem. Based on data from the Health Insurance Review and Assessment Service, domestic ultrasound imaging devices yield low-quality images [9]. An evaluation by the National Cancer Screening Agency indicated that the nonconformity rate for phantom imaging was 19.3%–23.3%, and the nonconformity rate for clinical imaging was 6.0%–45.7% [9]. Thus, it is necessary to reduce noise to enhance ultrasound image quality.

A representative method for improving image quality for ultrasound quality control is to employ software technologies. In particular, clutter and speckle noise in ultrasound images are common, and several studies on their removal have been conducted. Kang et al. observed that near-field clutter artifacts could be reduced using a wavelet transform in echocardiography [10]. Kim et al. confirmed that the image quality improved after noise filtering methods were applied to the thyroid ultrasound image in the spatial domain [11]. Although the image quality improvement efficiency of the wavelet transform and spatial filtering methods has been demonstrated, improved noise-reduction technology in diagnostic imaging is still being investigated. Among the noise-reduction methods, the non-local means (NLM) approach is modeled based on the weighting principle and effectively removes noise by measuring the similarity of local parts of an image [12]. Coupé et al. implemented the comparison study between NLM based speckle filtering and other methods in simulation and experiment [13]. Recently, the modified NLM method has also introduced to effectively remove the noise from ultrasound images in combination with deep-learning, which exhibits outperformed results [14,15].

In this study, we investigated that qualitative and clinical accuracy is improved when applying noise reduction algorithm in thyroid nodules. Here, the noise reduction algorithm is applied with a basic NLM method. The ATS 539 multipurpose phantom was employed, and a thyroid image was used to confirm its applicability in clinical practice.

## 2. Materials and Methods

### 2.1. Compliance with Ethical Standards

The study was conducted according to the guidelines of the Declaration of Helsinki and approved by the Institutional Review Board of Gachon University (1044396-202210-HR-218-01). Written consent was obtained from patients before they were included in the study.

### 2.2. Ultrasound Imaging System and Phantom

The probe used for scanning was a convex-type probe with 3.5 MHz frequency according to ATS regulations, and 30 images were captured and used for image analysis. An ATS-539 multipurpose phantom was used to scan the dynamic range and gray-scale measurement regions, which are most closely related to the noise level. In addition to the ATS phantom images, a human thyroid ultrasound image was also acquired. For the human ultrasound images, a linear probe with a frequency of 7.5 MHz was used.

### 2.3. NLM Noise-Reduction Algorithm Modeling

**Figure 1:**
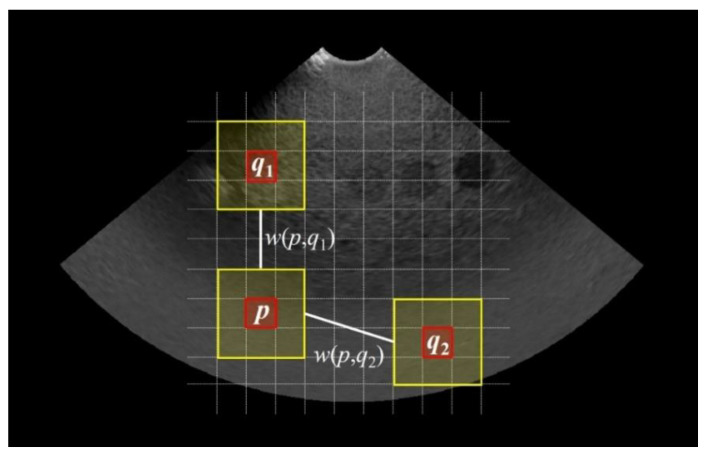
Description of weighting part during processing of NLM noise-reduction algorithm image.

In this study, the NLM noise-reduction approach was modeled using the method proposed by Buades et al. [16]. The NLM approach for basic noise reduction is based on Equation (1):

(1) $$I\left(i\right)=\sum_{j\in N_{i}}w_{ij}I\left(j\right)$$

where I(i) is the brightness of the ith pixel, $N_{i}$ is the search window value at the ith position, $w_{ij}$ is the weight value between the i and j pixels, and I(j) is the brightness of the jth pixel. The $w_{ij}$ representing the weight indicates the similarity between pixels i and j and should have a numerical value between 0 and 1. The final weight using the normalizing term, ($Z_{i}$), is defined as follows:

(2) $$w_{ij}=\frac{1}{Z_{i}}exp^{-\frac{\|\vec{I\left(i\right)}-\vec{I\left(j\right)}{\|}^{2}}{h^{2}}}$$

where h is a constant multiple of the standard deviation of the noise, and the degree of smoothing can be adjusted. A simple schematic of the weights is shown in Figure 1. Considering the p, $q_{1}$, and $q_{2}$ regions in the ultrasound sample image, the $q_{1}$ region exhibited highly consistent image similarity compared to the p region, but the $q_{2}$ region exhibited high heterogeneity. Based on these characteristics, noise could be reduced while maintaining sharpness by applying a large weight between the p and $q_{1}$ regions ($w\left(p,q_{1}\right)$) and a small weight between the p and $q_{2}$ regions ($w\left(p,q_{2}\right)$).

**Figure 2:**
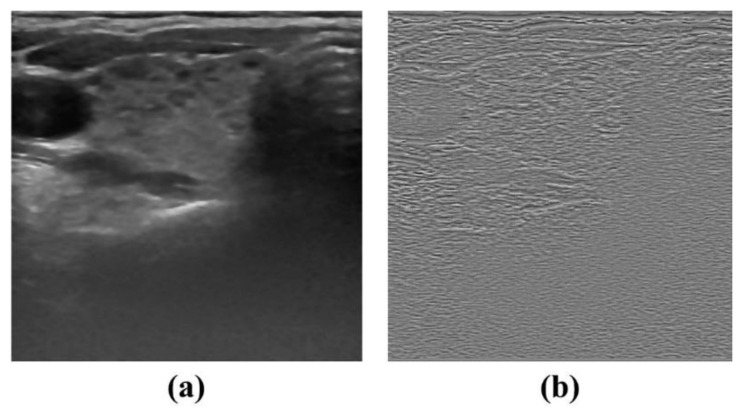
NLM denoising results using default standard deviation value (*δ* = 20) for (**a**) denoised ultrasound image and (**b**) residual image.

The weight was adjusted based on the Euclidean distance, and the standard deviation value (δ) was set to 20. Figure 2a,b show the original NLM denoised image and the corresponding residual image based on the default δ value, respectively.

### 2.4. Quantitative Evaluation of Image Quality

**Figure 3:**
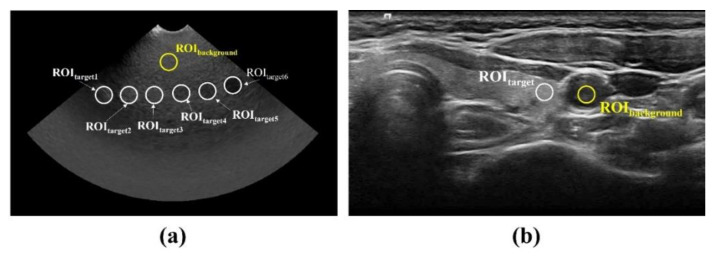
ROI setup for quantitative evaluation of ultrasound images: (**a**) ATS phantom and (**b**) human thyroid. In the ATS phantom image, the average values derived from six areas where gray-scale and dynamic range can be measured were used.

Parameters for evaluating the noise level were used to quantitatively analyze the applicability of the proposed NLM noise-reduction algorithm to ultrasound images. In this study, the noise level in ultrasound images was evaluated using the contrast-to-noise ratio (CNR) and coefficient of variation (COV). The CNR and COV values were calculated using Equations (3) and (4), respectively:

(3) $$\mathrm{CNR}=\frac{\left|S_{\mathrm{Target}}-S_{\mathrm{Background}}\right|}{\sqrt{\sigma_{\mathrm{Target}}{}^{2}+\sigma_{\mathrm{Background}}{}^{2}}}$$

(4) $$\mathrm{COV}=\frac{\sigma_{\mathrm{Target}}}{S_{\mathrm{Target}}}$$

where $S_{\mathrm{Target}}$ and $\sigma_{\mathrm{Target}}$ are the mean signal value and standard deviation of the region of interest (ROI) in the target, respectively, and $S_{\mathrm{Background}}$ and $\sigma_{\mathrm{Background}}$ are the mean signal value and standard deviation, respectively. Figure 3 shows the location of the ROI in the ATS phantom and human thyroid ultrasound images for the CNR and COV measurements.

Previously used filtering and denoising methods were modeled to verify the usefulness of the NLM noise-reduction algorithm. The noise-reduction efficiency of the ultrasound images modeled using the denoising methods was quantitatively analyzed based on the CNR and COV evaluation parameters.

## 3. Results

Quality control of a device used in diagnostic medical ultrasound is one of the most critical factors for improving the diagnostic accuracy of lesions. Reducing noise, which is essential when scanning an ultrasound image, helps achieve precise quality control. NLM-based algorithms, which are widely used in various diagnostic and medical imaging fields, are very effective. Thus, the purpose of this study was to model an NLM noise-reduction algorithm to verify its applicability to ultrasound images for quality control.

**Figure 4:**
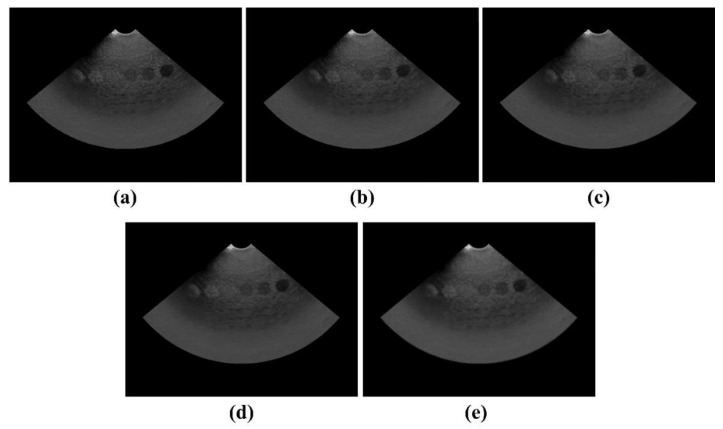
Acquired ATS ultrasound phantom images using (**a**) noisy, (**b**) median filter, (**c**) Wiener filter, (**d**) PGPD, and (**e**) proposed NLM noise-reduction algorithm.

**Figure 5:**
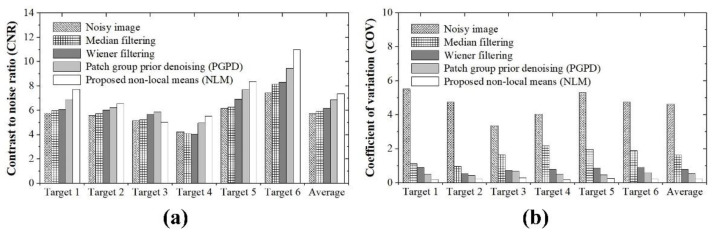
Graphs for quantitative evaluation of (**a**) CNR and (**b**) COV in acquired ATS ultrasound phantom images.

Figure 4 shows the ATS ultrasound phantom images acquired in the dynamic range and gray-scale evaluation area using the proposed NLM noise-reduction algorithm and conventional filtering methods. Through visual examination, we observed that the noise-reduction efficiency was best for the image to which the NLM algorithm was applied (Figure 4e). Two main ultrasound image quality metrics, that is, CNR and COV, were applied to the ATS phantom images. Figure 5a,b show the CNR and COV results, respectively, obtained by applying different conventional filtering methods, including the proposed NLM noise-reduction algorithm, to the acquired ATS phantom image. The average CNR and COV values were calculated by setting six ROI regions on the acquired ATS phantom images. The average CNR values obtained using the noisy image, median filter, Wiener filter, patch group prior denoising (PGPD), and proposed NLM noise-reduction algorithm were 5.73, 5.91, 6.17, 6.85, and 7.36, respectively. We confirmed that the CNR results for the ultrasound images subjected to NLM noise reduction improved by 28.62%, 24.66%, 19.37%, and 7.47% for the noisy image, median filter, Wiener filter, and PGPD, respectively. In addition, the average COV values obtained using the noisy image, median filter, Wiener filter, PGPD, and proposed NLM noise-reduction algorithm were 4.63, 1.63, 0.79, 0.53, and 0.24, respectively. The CNR values of the ultrasound images for the noisy image, median filter, Wiener filter, and PGPD subjected to NLM noise reduction improved 19.54, 6.89, 3.34, and 2.25 times, respectively.

**Figure 6:**
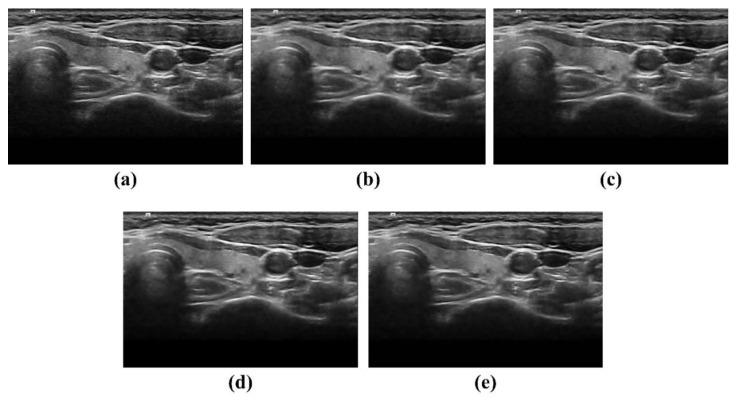
Acquired human thyroid ultrasound images captured using (**a**) noisy, (**b**) median filter, (**c**) Wiener filter, (**d**) PGPD, and (**e**) proposed non-local means noise-reduction algorithm.

**Figure 7:**
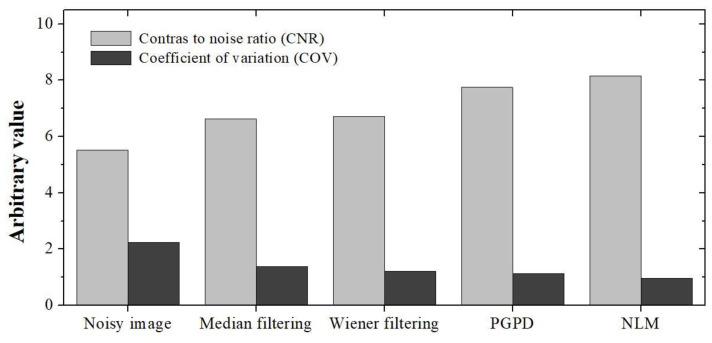
Graphs for quantitative evaluation of CNR and COV in acquired human thyroid ultrasound images.

Figure 6 shows the results of applying noise-reduction methods to an actual thyroid ultrasound image. When the NLM noise-reduction algorithm was applied, the noise level was visually reduced the most, and the trend was similar to the ATS phantom results. In particular, when the NLM noise-reduction algorithm was applied to the resulting ultrasound image, the noise that could lead to misdiagnosis of the disease was significantly reduced in the parenchyma of the thyroid lobe. The CNR and COV were evaluated to objectively quantify the noise-reduction level by setting the thyroid lobe part as a target and the peripheral blood vessel part as a background. Figure 7 shows the CNR and COV results obtained by applying different filtering methods, including the proposed NLM noise-reduction algorithm, to the acquired thyroid ultrasound image. The CNR values for thyroid ultrasound images were 5.52, 6.64, 6.71, 7.75, and 8.15 for the noisy image, median filter, Wiener filter, PGPD, and proposed NLM noise-reduction algorithm, respectively. The CNR values for the ultrasound images subjected to NLM noise reduction improved by 47.64%, 22.74%, 21.46%, and 5.16% for the noisy image, median filter, Wiener filter, and PGPD, respectively. In addition, the COV values were 4.625, 1.630, 0.790, 0.532, and 0.237 for the noisy image, median filter, Wiener filter, PGPD, and proposed NLM noise-reduction algorithm for thyroid ultrasound images, respectively. The CNR values of the ultrasound images subjected to NLM noise reduction improved 2.36, 1.44, 1.28, and 1.18 times for the noisy image, median filter, Wiener filter, and PGPD, respectively.

## 4. Discussion

For optimal operation of the diagnostic imaging system, including ultrasonography, the user should periodically inspect and check for defects in the device [16]. In Korea, quality control for computed tomography, magnetic resonance imaging, and mammography was implemented in 2004, centered on the Korea Medical Imaging Quality Management Institute. In particular, phantom and actual clinical images have been inspected for image quality, including the management of equipment and personnel for these systems. However, the legalization of quality control for ultrasound imaging devices is yet to be actively implemented, and many difficulties in quality control are encountered owing to inappropriate image quality, necessitating system improvement. We developed an NLM noise-reduction approach that is more efficient than existing approaches in controlling ultrasonic picture quality. In addition, we verified the applicability of the proposed algorithm using an area capable of measuring the dynamic range and gray scale of the ATS phantom.

A significant drawback of lesion diagnosis using an ultrasound image is that the diagnostic accuracy depends on the user’s ability to scan the image. Hence, maintaining the image quality is critical. In addition, because the intrinsic characteristics of the patient significantly influence the ultrasound image, it is essential to control the quality of the device and maintain image quality. Thus, objective indicators must be set to overcome these problems, and quantitative image quality evaluation is expected to improve ultrasound quality control. Among the image qualities, noise is one of the most significant factors that reduces the accuracy of lesion diagnosis in ultrasound images. An approach is required for denoising and laying the foundation for quality control measures for noise levels [17].

Noise in an ultrasound image can be generated in various forms. Speckle noise, the most critical type of noise for degrading ultrasound image quality, is a product type in which the mean and standard deviation are mutually proportional and generally follow a Rayleigh distribution [18]. Improved median and Wiener filters have been widely used to reduce speckle noise generated in ultrasound images [19,20]. However, due to difficulties in processing the generated noise assuming a Gaussian distribution or when the mean value and variance of the image are proportional, many parts undesirable for the ultrasound image appear. In addition, Gupta et al. modeled various filters and analyzed the speckle noise suppression efficiency in ultrasound images [21]. However, none of the previously developed filters has high noise-removal efficiency for ultrasound images, and a patch group-based filtering method has been used to compensate for these shortcomings. PGPD is a patch group-based filtering method, and it is modeled using a method that adequately removes noise through the learning and denoising stages [22]. The learning stage of PGPD is a process of grouping similar patch types. During the denoising stage, noise is appropriately reduced using various control parameters for the learned patch group. Kim et al. modeled this principle as a filter and applied PGPD to simulations and actual thyroid ultrasound images; through various quantitative evaluations, they proved that it exhibits superior characteristics compared to conventional noise-removal methods [11]. In this study, we developed an adequate NLM approach with higher noise-removal efficiency than PGPD, whose superiority has been demonstrated. When the proposed NLM noise-reduction algorithm was applied to human thyroid ultrasound images, the CNR and COV improved by 5.16% and 1.18 times, respectively, compared to PGPD.

Blurring inevitably occurs when software technology is applied to remove noise from ultrasound images. Such blurring of the ultrasound image deteriorates the edge information, affecting diagnostic accuracy. Thus, the noise level and the degree of blurring should be determined simultaneously to assess ultrasound image quality. Several methods for evaluating the overall quality of medical images are available, but recently, parameters that can be evaluated without a reference image were proposed by Mittal et al. [23,24]. Representative methods of image quality assessment based on no-reference include the blind/referenceless image spatial quality evaluator (BRISQUE) and the natural image quality evaluator (NIQE). Although both evaluation methods are models developed based on natural science statistics, NIQE is more accurate than BRISQUE because it can satisfactorily reflect the corpus of natural images [24]. Thus, we evaluated NIQE to measure the noise level and the degree of blurring in the captured human thyroid ultrasound image. When the NLM noise-reduction algorithm was applied to the ultrasound image, a NIQE of 16.93 was obtained. When the noisy image, median filter, Wiener filter, and PGPD were used, the NIQE values were 16.68, 17.11, 17.29, and 17.06, respectively. Based on the NIQE results, we can infer that when the NLM noise-reduction algorithm is applied to the ultrasound image, the edge part closest to the noisy image can be preserved. Compared to conventional denoising methods, the NLM noise-reduction algorithm produces excellent quality of the entire ultrasound image. In an ultrasound image, the edge area should be adequately maintained for accurate length measurements of lesions and human organs. The proposed NLM noise-reduction algorithm can reduce the signal distortion and blurring effect of conventional filtering methods in terms of the overall geometric structure of the image [25].

**Figure 8:**
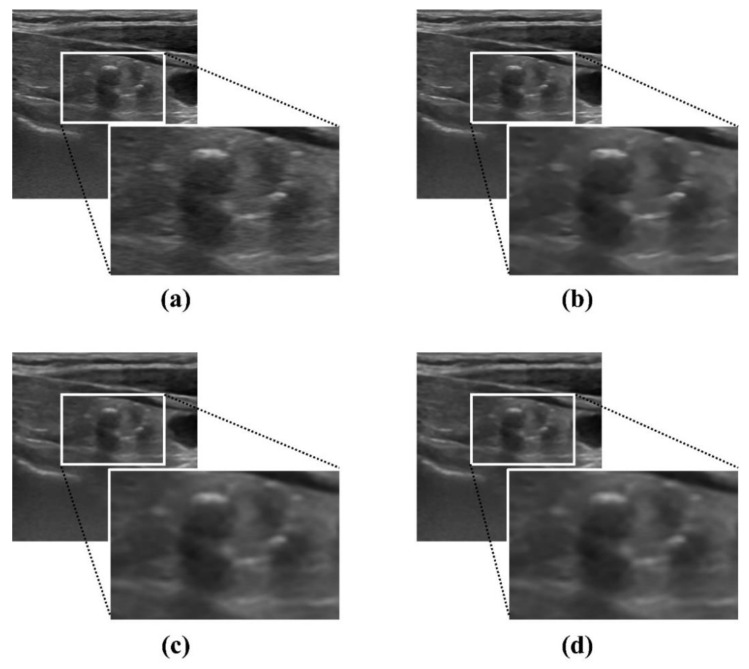
Ultrasound images of patients with thyroid nodule: (**a**) noisy image and NLM denoising with (**b**) low smoothing, (**c**) middle smoothing, and (**d**) high smoothing.

**Figure 9:**
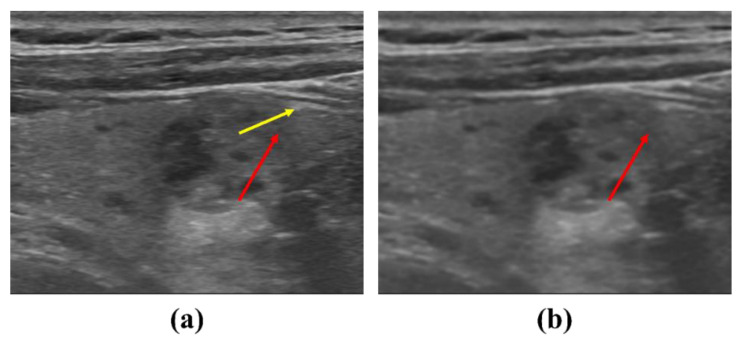
Ultrasound images to observe changes in the margin of the thyroid nodule: (**a**) noisy image and (**b**) NLM denoising with high smoothing. The red arrow means the actual exact nodule margin, and the yellow arrow means the margin that can be misunderstood.

The use of proposed NLM noise-reduction algorithm in diagnostic ultrasound imaging is expected to help diagnose thyroid diseases and increase the diagnostic efficiency. Figure 8 shows the results for the proposed NLM noise-reduction algorithm applied to an ultrasound image of a thyroid nodule patient after each smoothing step. The lesion was visually observed in all stages of low, middle, and high smoothing using the NLM noise-reduction algorithm. In addition, the proposed algorithm may be useful when the diagnosis is difficult since the margin is unclear when following up on the changes in the thyroid mass size. The margin part was observed more clearly when the high smoothing step was applied to the thyroid ultrasound image than with the low smoothing step, and we expected that the size measurement accuracy of the mass part would be improved. Figure 9 is an ultrasound image of the thyroid nodule in order to observe in more detail the margin change with and without algorithm application (red arrow: margin of actual nodule, yellow arrow: part that can be mistaken for margin). As shown in Figure 9a, it can be seen that the margin of the thyroid nodule is not clear in the original ultrasound image before the denoising process. However, in Figure 9b, when the NLM noise-reduction algorithm based on high smoothing is used, we can confirm that the margin is relatively clearly revealed.

In particular, the shape, size, and degree of echo of thyroid nodules observed on ultrasound images vary widely; therefore, a framework that satisfies Korean standards was established, called the “Korean Thyroid Imaging Reporting and Data System (K-TIRADS)” [26]. The K-TIRADS is divided into five stages based on the clinical observations of the ultrasound image, and the probability of malignancy and biopsy criteria are presented. Among nodule types, approximately 3% of spongiform nodules are likely malignant, and biopsy is recommended if they are larger than 2 cm. In addition, when a nodule is classified into four stages, a maximum 50% chance of malignancy exists; therefore, if the nodule exceeds 1 cm, a biopsy should be performed immediately. We expect that the proposed algorithm will help measure the exact size of a thyroid nodule and determine whether biopsy is indicated or not.

Recently, researchers have actively investigated the segmentation of organs or structures in ultrasound images using deep-learning and image-processing techniques. Hafiane et al. used the convolutional neural network (CNN) architecture, which is the most widely used deep-learning technique, and the neural part of the ultrasound image was separated more precisely than with conventional methods [27]. In addition, a fusion model of a CNN and recurrent neural network in the ultrasound field was established by Chen et al., and the generative adversarial network was confirmed to be a very effective model for segmentation [28,29]. During this segmentation process, the noise from the ultrasound image increases the false positive rate, reducing the accuracy of the deep-learning algorithm. If the proposed NLM noise-reduction algorithm is applied to deep-learning-based ultrasound imaging technology, it is expected that the accuracy and usefulness of the deep-learning model will improve. In addition, the proposed algorithm applies not just to the thyroid area, but to a technique that can improve ultrasound imaging accuracy for abdominal diseases and reduce mirror artifacts.

## 5. Conclusions

In this work, we evaluated the image performance and accuracy of diagnostic according to the speckle noise reduction using the NLM algorithm in ultrasound thyroid image. The NLM noise-reduction algorithm yields better metrics than conventional denoising methods. The accuracy of clinical ultrasound quality control can be improved using the results of this study, and the misdiagnosis rate of lesions in organs, including the thyroid gland, can be minimized.

## Data Availability

Not applicable.
